# Supplementary figures and images for: Coupling between spatial compartments integrates morphogenetic patterning in the organ of Corti
Source: PLoS Biol. 2025 Sep 9;23(9):e3003350. doi: 10.1371/journal.pbio.3003350 (PMC12419656; doi:10.1371/journal.pbio.3003350)

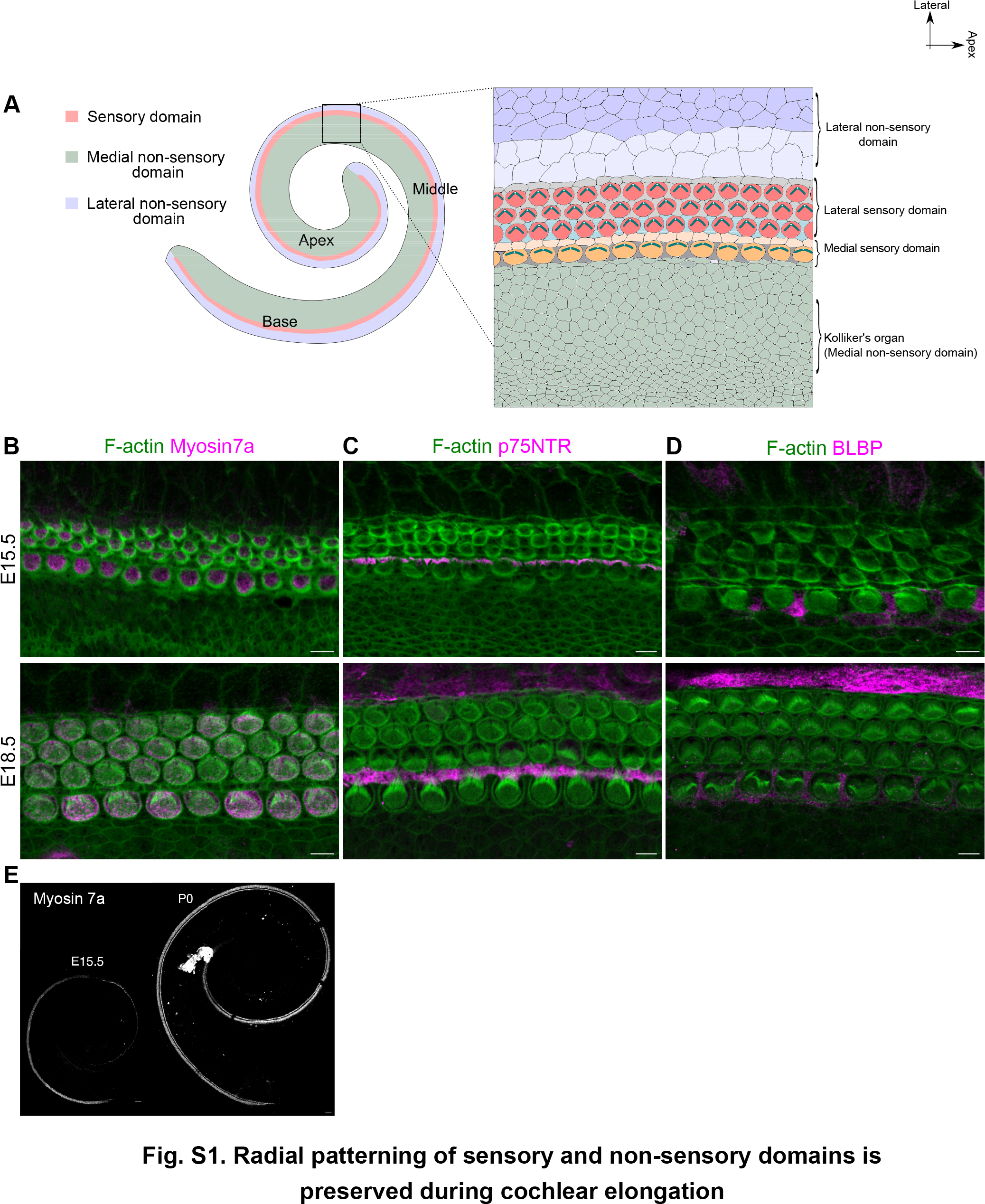

Supplement: S1 Fig — (A) Schematic representing the organization of various sensory and non-sensory domains and their constituent cell types in the mouse organ of Corti. (B) Base of E15.5 and E18.5 OC stained for F-actin (green) and Myosin7a (magenta). (C) Base of E15.5 and E18.5 OC stained for F-actin (green) and p75NTR (magenta). (D) Base of E15.5 and E18.5 OC stained for F-actin (green) and BLBP (magenta). (E) OC from E15.5 and PO stained for Myosin 7a. Scale Bar: 50 µm in E and 5 µm in B–D. Image orientation: Top is lateral, Right is Apex. (TIF) [file pbio.3003350.s001.tif]

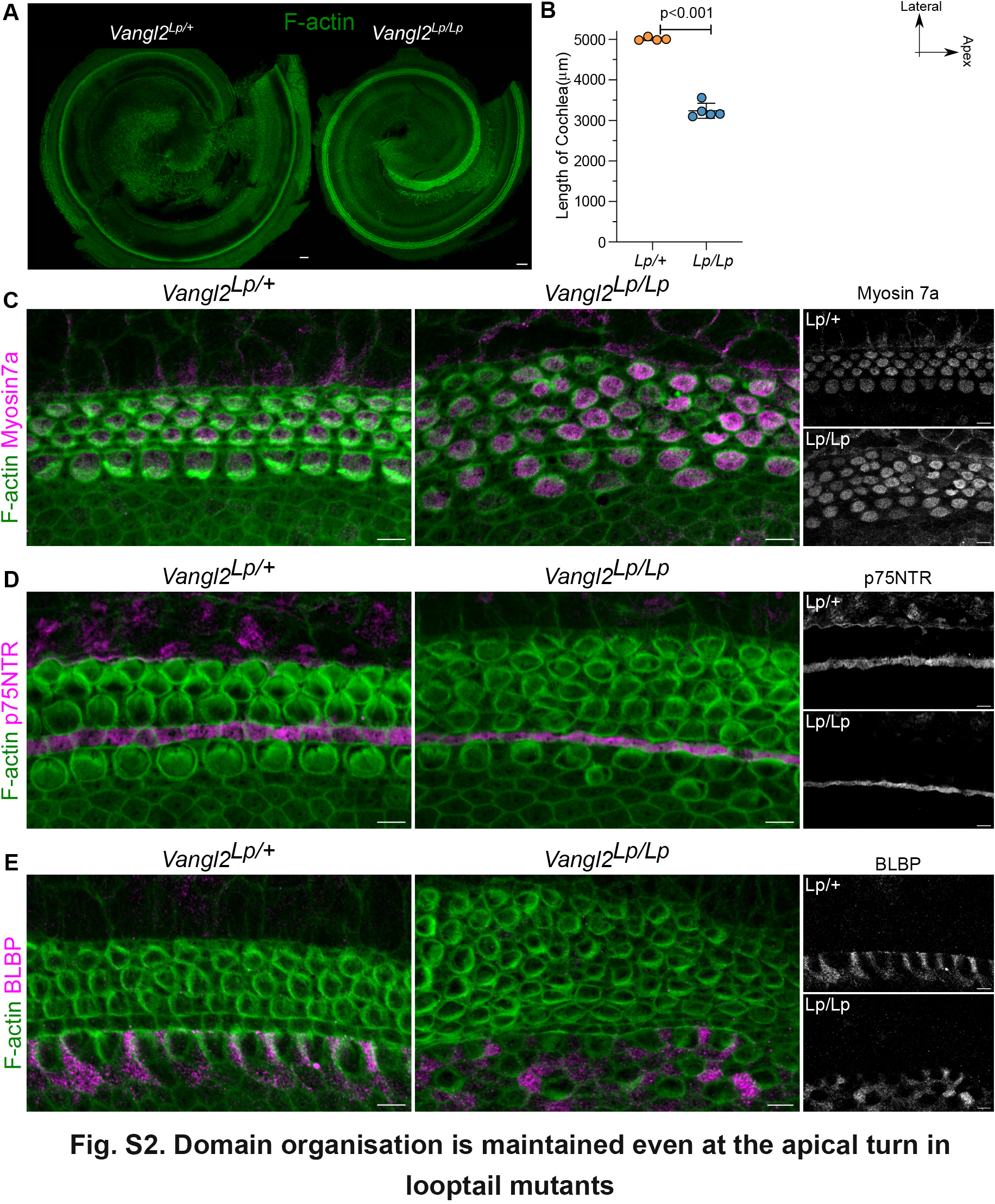

Supplement: S2 Fig — (A) OC from embryonic day (E)18.5 heterozygous (Vangl2 Lp/+) and homozygous (Vangl2 Lp/Lp) looptail mutant stained for f-actin (green). (B) Length of E18.5 cochlea from heterozygous (Vangl2 Lp/+) and homozygous (Vangl2 Lp/Lp) looptail mutant. N = 4/ 5 (Het/Homo). (C) Apex of E18.5 OC from heterozygous (Vangl2 Lp/+) and homozygous (Vangl2 Lp/Lp) looptail mutant stained for F-actin (green) and Myosin 7a (magenta and gray). N = 4. (D) Apex of E18.5 OC from heterozygous (Vangl2 Lp/+) and homozygous (Vangl2 Lp/Lp) looptail mutant stained for F-actin (green) and p75NTR (magenta and gray). N = 4. (E) Apex of E18.5 OC from heterozygous (Vangl2 Lp/+) and homozygous (Vangl2 Lp/Lp) looptail mutant stained for F-actin (green) and BLBP (magenta and gray). N = 4. Scale Bar: 50 µm in B and 5 µm in C–E. Unpaired T test. Image orientation: Top is lateral, Right is Apex. Underlying data available in S1 Data (TIF) [file pbio.3003350.s002.tif]

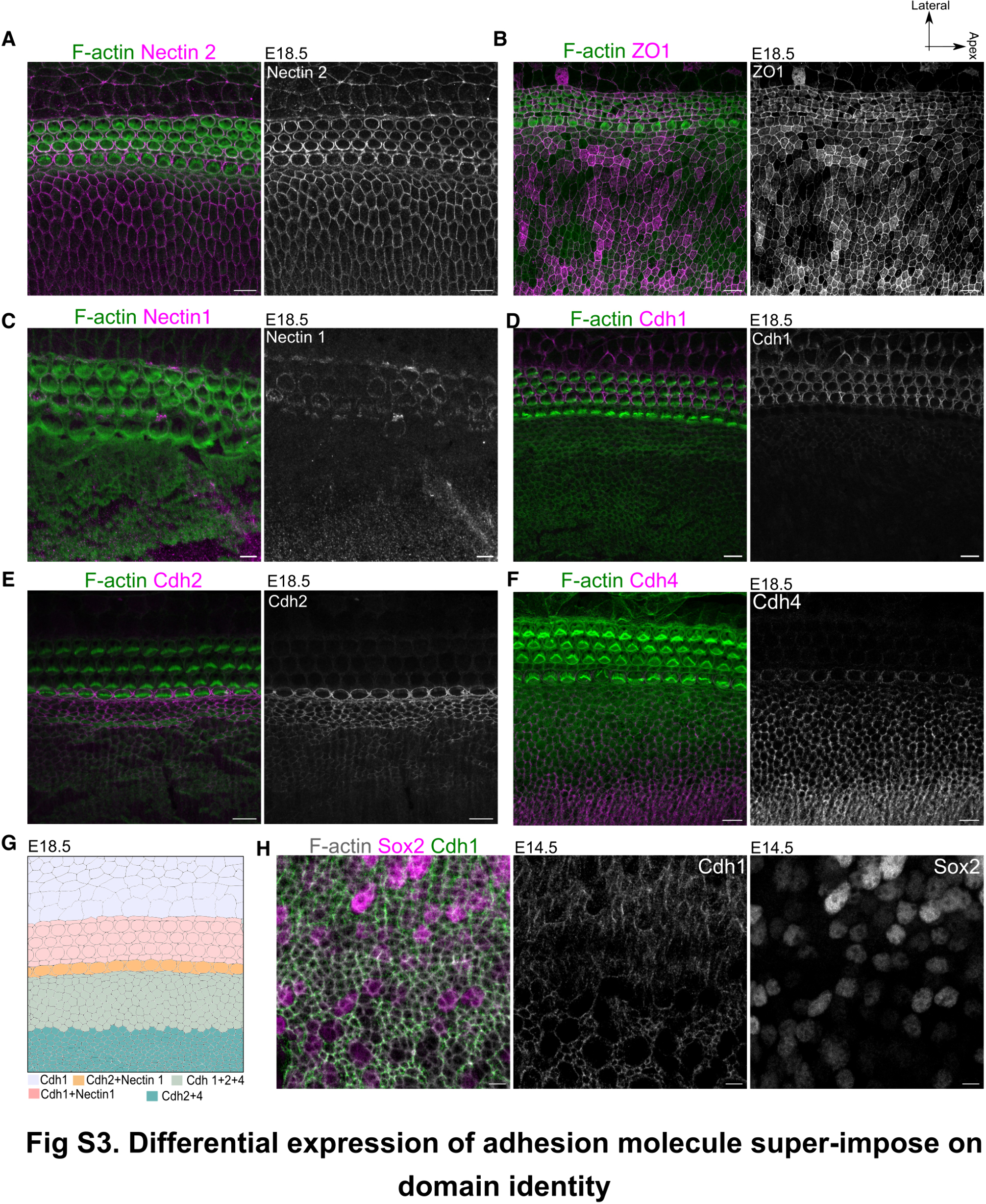

Supplement: S3 Fig — (A) E18.5 OC stained for F-actin (green) and Nectin2 (magenta and gray). (B) E18.5 OC stained for F-actin (green) and Zonula Occludens-1 (ZO1)(magenta and gray). (C) E18.5 OC stained for F-actin (green) and Nectin2 (magenta and gray). (D) E18.5 OC stained for F-actin (green) and Cdh1 (magenta and gray). (E) E18.5 OC stained for F-actin (green) and Cdh2 (magenta and gray). (F) E18.5 OC stained for F-actin (green) and Cdh4 (magenta and gray). (G) Schematic representing the combinatorial expression of adhesion molecule super-imposed on the cell types of OC. (H) E14.5 OC stained for F-actin (gray), Sox2 (magenta and gray), and Cdh1 (green and gray). Scale Bar: 10 µm. Image orientation: Top is lateral, Right is Apex. (TIF) [file pbio.3003350.s003.tif]

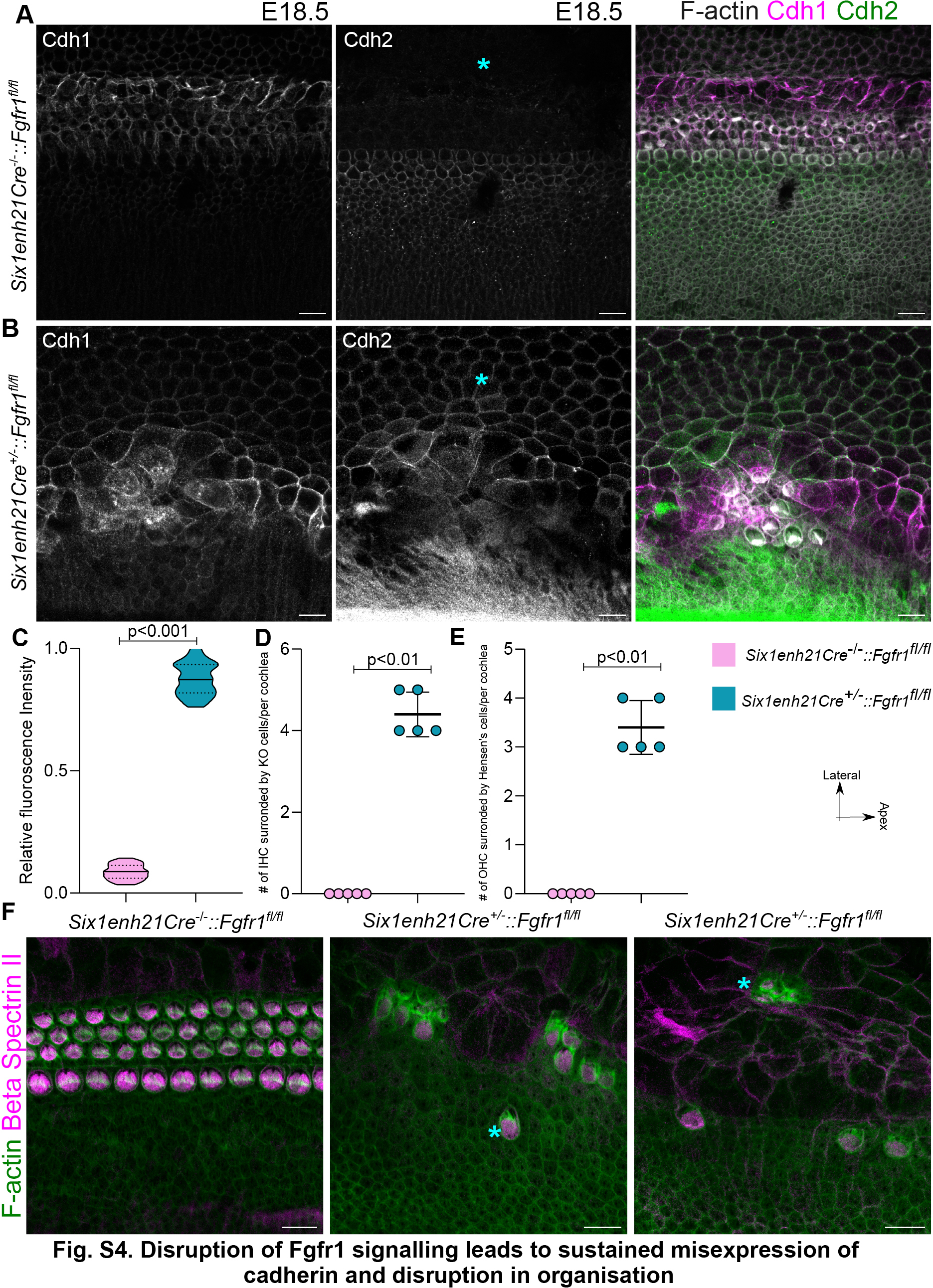

Supplement: S4 Fig — (A) E18.5 OC from control embryos (Six1enh21Cre−/−::Fgfr1fl/fl) stained for Cdh1 (gray and magenta), Cdh2 (gray and green), and F-actin (gray in merged). Asterisk indicates the absence of Cdh2 signals from lateral non-sensory domain. N = 4 embryos. (B) E18.5 OC from Fgfr1 mutant embryos (Six1enh21Cre+/−::Fgfr1fl/fl) stained for Cdh1 (gray and magenta), Cdh2 (gray and green), and F-actin (gray in merged). Asterisk indicates the ectopic Cdh2 signals from lateral non-sensory domain. N = 4 embryos. (C) Relative Fluorescence Intensity of Cdh2 in Claudius cells in control and Fgfr1 mutant cochlea at E18.5. (D) Number of IHC surrounded by KO cells on all side per cochlea in control and fgfr1 mutant cochlea. This shows medial non-sensory domain is intermixed with the medial sensory domain. (E) Number of OHC surrounded by Hensen’s cells on all side per cochlea in control and fgfr1 mutant cochlea. This shows lateral non-sensory domain is intermixed with the lateral sensory domain. (F) E18.5 OC from control and Fgfr1 mutant embryos (Six1enh21Cre+/−::Fgfr1fl/fl) stained for F-actin (green) and HC marker Beta Spectrin II (magenta). Asterisk indicates the presence of HCs in the lateral and medial non-sensory domains. N = 5 embryos. Unpaired T test. Scale Bar: 10 µm. Image orientation: Top is lateral, Right is Apex. Underlying data available in S1 Data. (TIF) [file pbio.3003350.s004.tif]

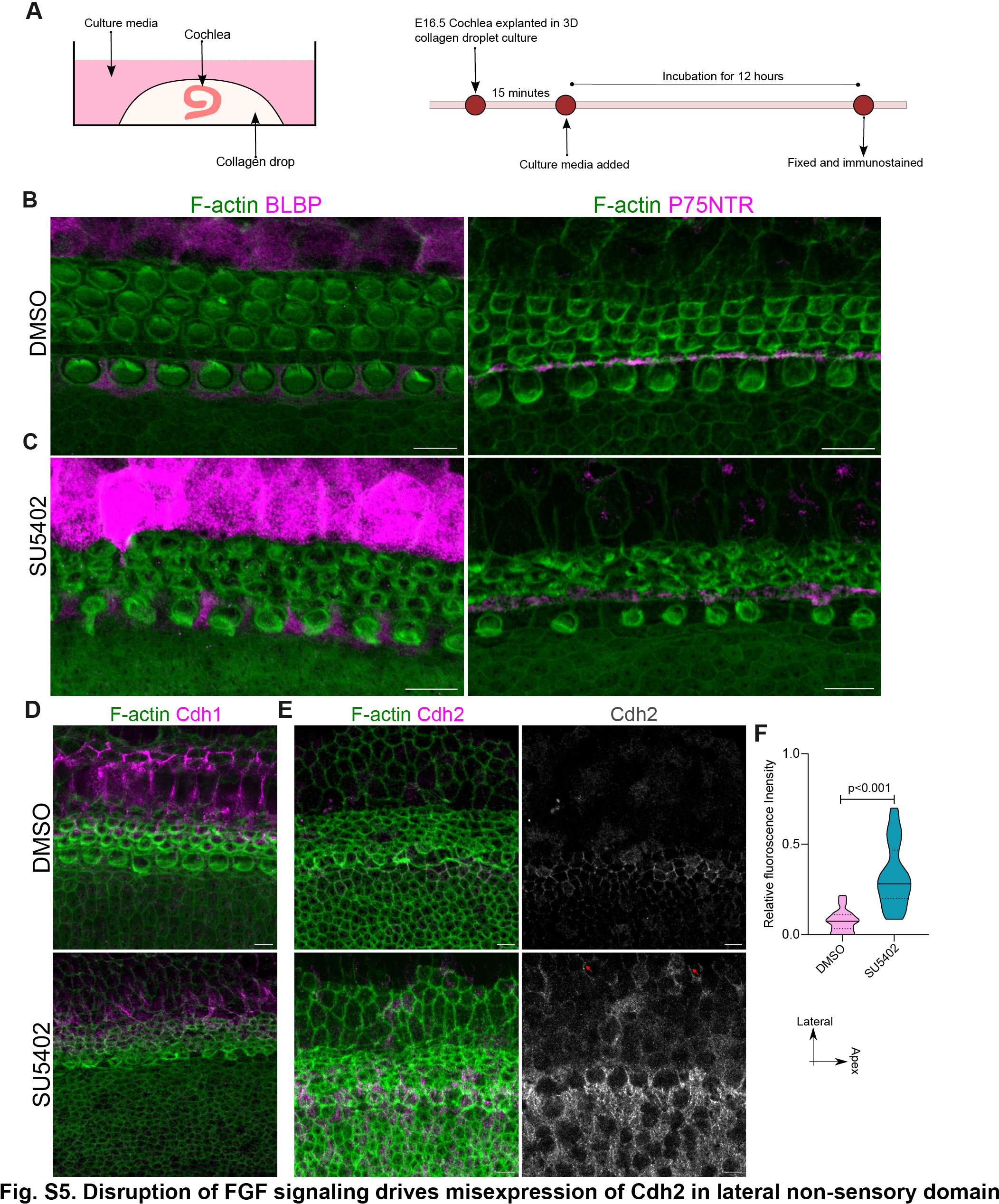

Supplement: S5 Fig — (A) Schematic representing the collagen droplet culture and treatment condition for an ex vivo explant culture of OC. (B) E16.5 Cochlea cultured in presence of DMSO for 12 h stained for F-actin (green), BLBP (magenta), and p75NTR (magenta). (C) E16.5 Cochlea cultured in presence of Fgfr1 inhibitor (Su5402, 25 µM) stained for F-actin (green), BLBP (magenta), and p75NTR (magenta). (D) E16.5 Cochlea cultured in presence of Fgfr1 inhibitor (Su5402, 25 µM) stained for F-actin (green), Cdh1(magenta). (E) E16.5 Cochlea cultured in presence of Fgfr1 inhibitor (Su5402, 25 µM) stained for F-actin (green), Cdh2 (magenta, gray) showing misexpression of Cdh2 in Claudius cells similar to the genetic perturbation in S4B Fig. (F) Relative Fluorescence Intensity of Cdh2 in Claudius cells in control (DMSO treated) and Fgfr1 inhibited (Su5402 treated) cochlea at E18.5. Unpaired T test. Scale Bar: 10 µm. Image orientation: Top is lateral, Right is Apex. Underlying data available in S1 Data. (TIF) [file pbio.3003350.s005.tif]

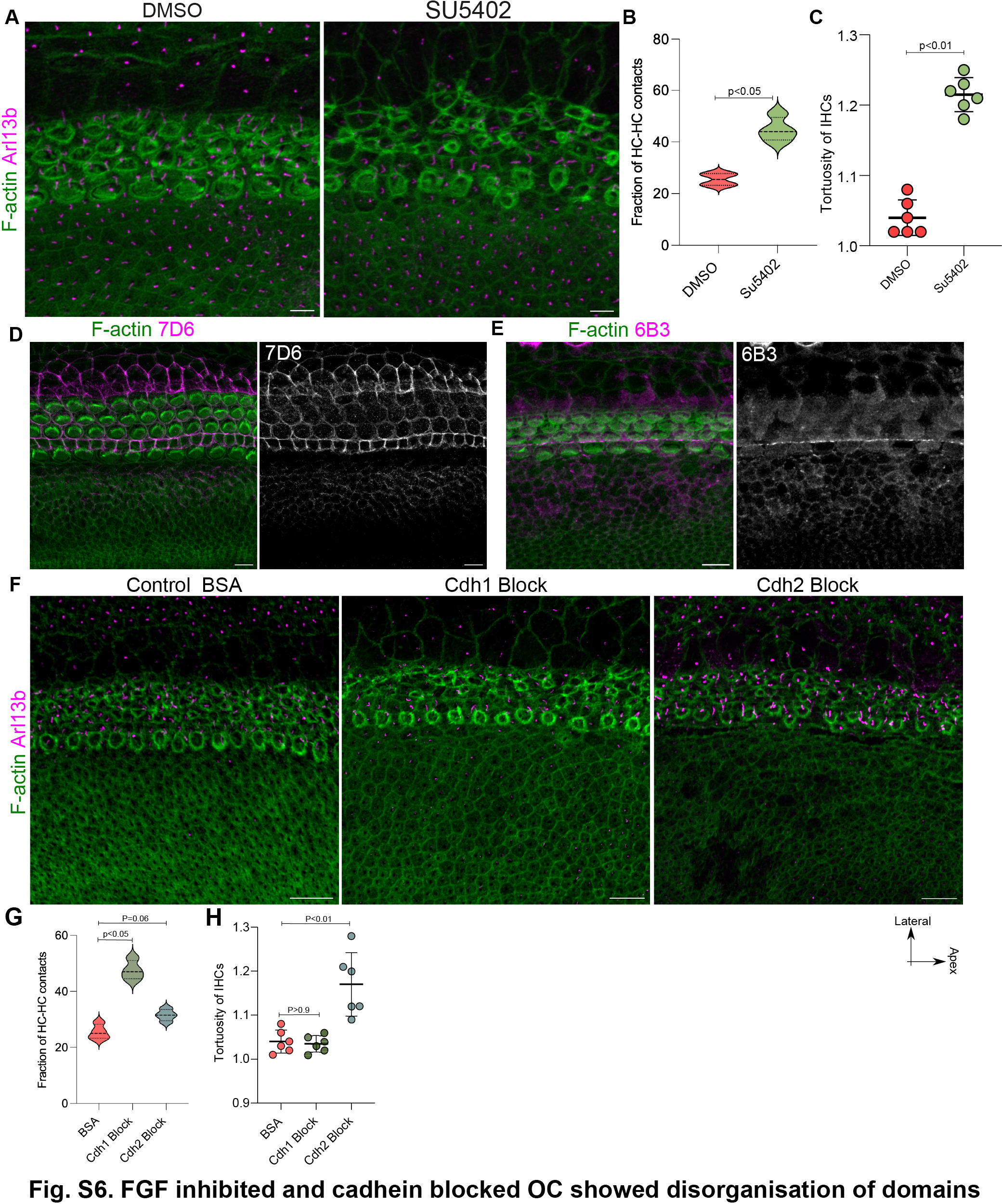

Supplement: S6 Fig — (A) E16.5 Cochlea cultured in presence of DMSO and Su5402 for 12 h stained for F-actin (green), BLBP (magenta). (B) Fraction of HC-HC contacts in DMSO-treated and Su5402-treated OC. (C) Straightness of IHC represented by low tortuosity in DMSO-treated cochlea and the disruption of this straightness represented by high tortuosity in Su5402-treated cochlea. (D) Cochlea cultured for 1 h in presence of cadherin blocking antibodies 7D6, which block interactions among Cdh1, stained using F-actin (green) and secondary antibodies (magenta, gray). (E) Cochlea cultured for 1 h in presence of cadherin blocking antibodies 6B3, which block interactions among Cdh2, stained using F-actin (green) and secondary antibodies (magenta, gray). (F) 12-h explant of E15.5 OC in presence of Bovine Serum Albumin (BSA, 0.1%), Cdh1 blocking antibodies (7D6, 10 µg/ml), Cdh2 blocking antibodies (6B3, 10 µg/ml) stained for F-actin (green) and Arl13b (magenta). N = 4 cochlea. (G) Fraction of HC-HC contacts in BSA-treated, Cdh1-blocked, and Cdh2-blocked OC. (H) Tortuosity of IHC in BSA-treated, Cdh1 blocked and Cdh2-blocked OC. Unpaired T test. Scale Bar: 10 µm and Image orientation: Top is lateral, Right is Apex. Underlying data available in S1 Data. (TIF) [file pbio.3003350.s006.tif]

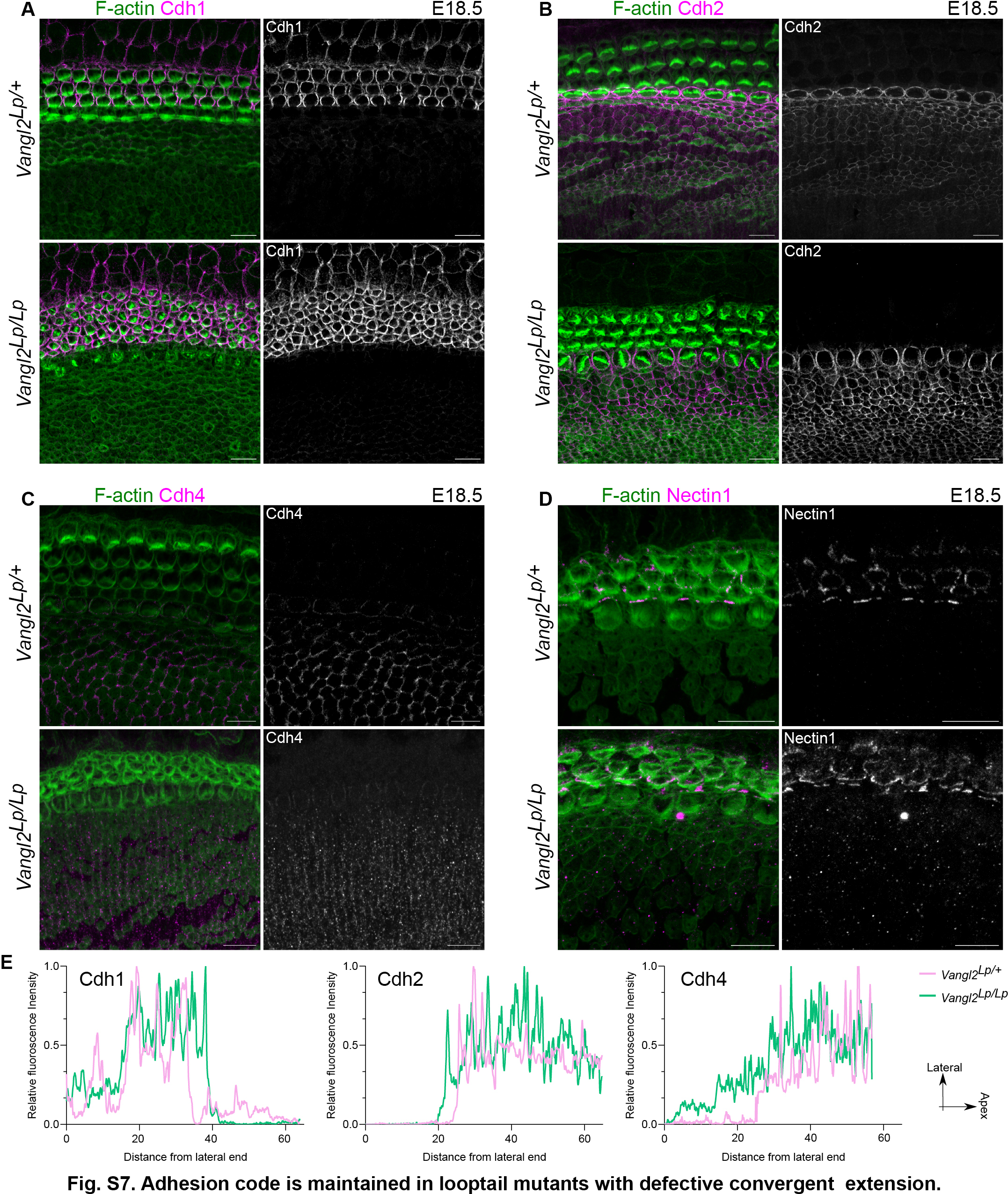

Supplement: S7 Fig — (A) E18.5 OC from heterozygous (Vangl2 Lp/+) and homozygous (Vangl2 Lp/Lp) looptail mutant stained for F-actin (green) and Cdh1 (magenta and gray). N = 3 cochlea. (B) E18.5 OC from heterozygous (Vangl2 Lp/+) and homozygous (Vangl2 Lp/Lp) looptail mutant stained for F-actin (green) and Cdh2 (magenta and gray). N = 3 cochlea. (C) E18.5 OC from heterozygous (Vangl2 Lp/+) and homozygous (Vangl2 Lp/Lp) looptail mutant stained for F-actin (green) and Cdh4 (magenta and gray). N = 3 cochlea. (D) E18.5 OC from heterozygous (Vangl2 Lp/+) and homozygous (Vangl2 Lp/Lp) looptail mutant stained for F-actin (green) and Nectin1 (magenta and gray). N = 3 cochlea. (E) Relative fluorescence intensity of Cdh1, Cdh2, and Cdh4 along the medio-lateral axis of OC at E18.5 from heterozygous (Vangl2 Lp/+) and homozygous (Vangl2 Lp/Lp) looptail mutant in pink and green, respectively. Scale Bar: 10 µm. Image orientation: Top is lateral, Right is Apex. Underlying data available in S1 Data (TIF) [file pbio.3003350.s007.tif]

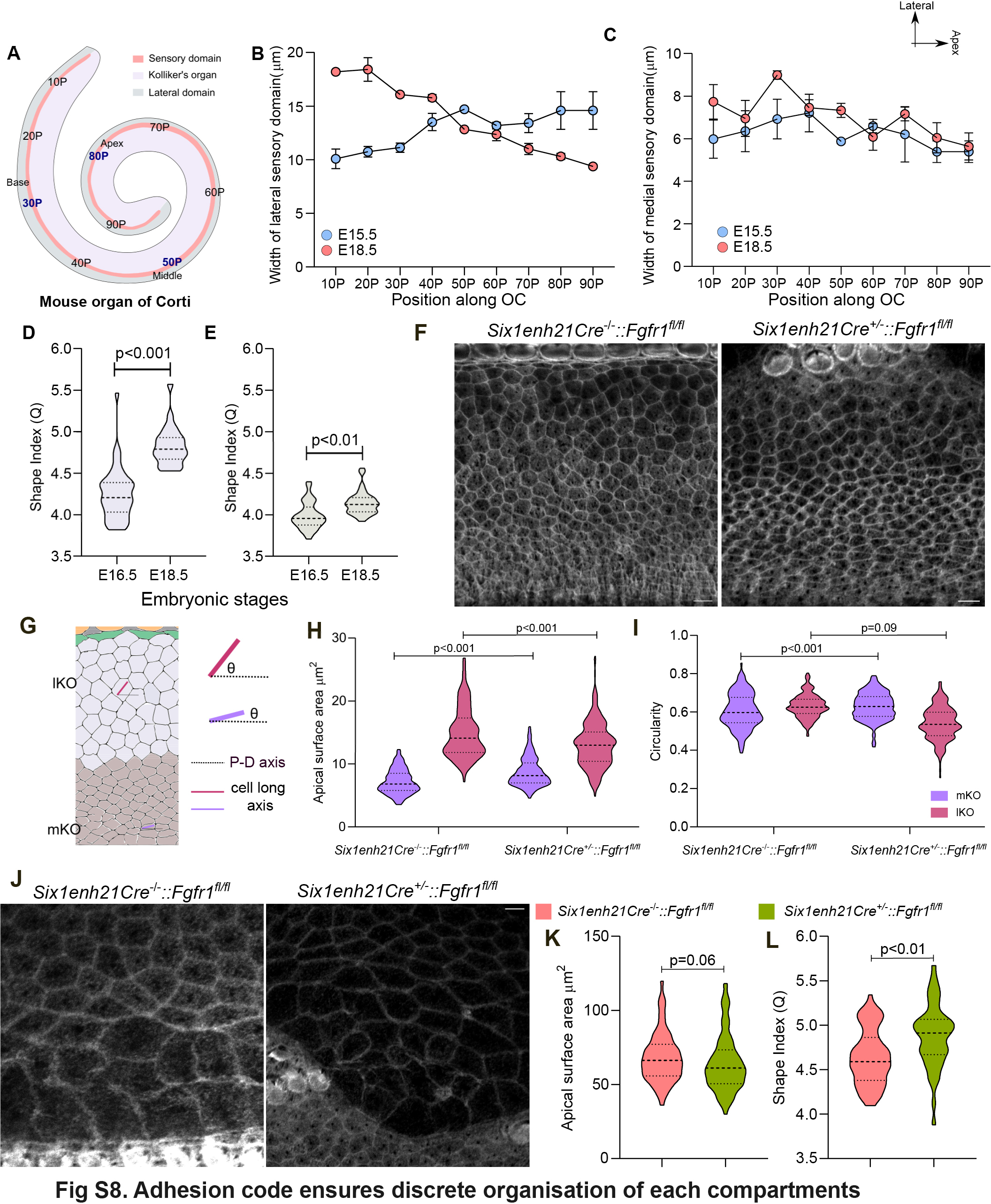

Supplement: S8 Fig — (A) Schematic of OC representing the nine equidistant points along the base-apex axis of the OC. (B) Width of the lateral sensory domain along the nine positions along the base-apex axis at E15.5 and E18.5. N = 4 cochlea each stage. (C) Width of the medial sensory domain along the nine positions along the base-apex axis at E15.5 and E18.5. N = 4 cochlea each stage. (D) Shape index (Q = perimeter/sqrt of area) of Hensen’s Cells at E16.5 and E18.5. N = 150/169 for Hensen and 144/153 for Claudius (E16.5/E18.5). (E) Shape index (Q = perimeter/sqrt of area) of Claudius Cells at E16.5 and E18.5. N = 144/153 (E16.5/E18.5). (F) E18.5 base of OC from control (Six1enh21Cre−/−::Fgfr1fl/fl) and Fgfr1 mutant embryos (Six1enh21Cre+/−::Fgfr1fl/fl) stained for F-actin (gray) showing KO domain. N = 4. (G) Schematic representing the calculation of the axis of cell elongation in mKO and lKO cells of KO domain. (H) Apical surface area of mKO and lKO cells from control (Six1enh21Cre−/−::Fgfr1fl/fl) and Fgfr1 mutant embryos (Six1enh21Cre+/−::Fgfr1fl/fl). N = 150/216 for control and 209/262 for mutant (mKO/lKO). (I) Circularity of mKO and lKO cells from control (Six1enh21Cre−/−::Fgfr1fl/fl) and Fgfr1 mutant embryos (Six1enh21Cre+/−::Fgfr1fl/fl). N = 150/216 for control and 209/262 for mutant (mKO/lKO). (J) E18.5 base of OC from control (Six1enh21Cre−/−::Fgfr1fl/fl) and Fgfr1 mutant embryos (Six1enh21Cre+/−::Fgfr1fl/fl) stained for F-actin (gray) showing lateral non-sensory domain. N = 4. (K) Apical surface area of Hensen’s Cells from control (Six1enh21Cre−/−::Fgfr1fl/fl) and Fgfr1 mutant embryos (Six1enh21Cre+/−::Fgfr1fl/fl). N = 65/98. (control/mutant). (L) Shape index of Hensen’s Cells from control (Six1enh21Cre−/−::Fgfr1fl/fl) and Fgfr1 mutant embryos (Six1enh21Cre+/−::Fgfr1fl/fl). N = 65/98. (control/mutant). Scale Bar: 10 µm. Unpaired T test. Image orientation: Top is lateral, Right is Apex. Six1enh21Cre−/− means cre negative and Six1enh21Cre+/− cre positive. Underlying data availabl [file pbio.3003350.s008.tif]

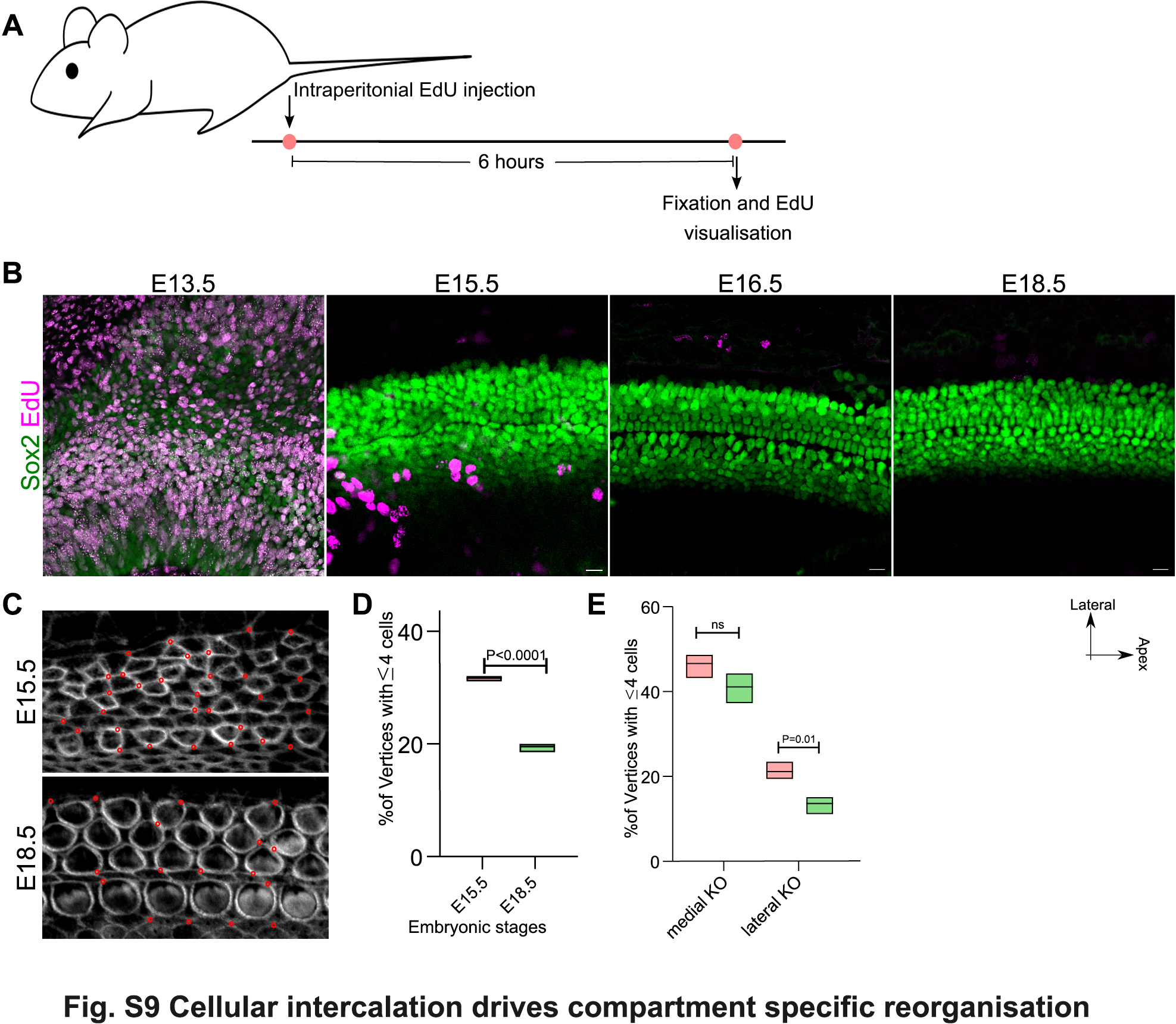

Supplement: S9 Fig — (A) Schematic representing the timeline of EdU injection and staining. (B) OC of embryos from E13.5, E15.5, E16.5, and E18.5 pregnant females injected with EdU stained for Sox2 (green) to mark sensory epithelia and click-chemistry based EdU (Magenta). N = 3. (C) OC from E15.5 and E18.5 base position (30P) stained for F-actin (gray), overlayed with red circles representing vertex with 4 or more cells. (D) Percentage of vertex with 4 or more cells in the sensory domain at E15.5 and E18.5. N = 3 embryos each with 205/615 for E15.5 and 122/625 for E18.5 (4 or more cell vertices/Total vertices). (E) Percentage of vertex with 4 or more cells in the medial and lateral KO domain at E15.5 and E18.5. N = 3 cochlea each, 498/1070 mKO at E15.5 and 259/630 at E18.5; 318/1532 lKO at E15.5 and 111/835 at E18.5 (4 or more cell vertices/Total vertices). Scale Bar: 20 µm for E13.5 and 10 µm for rest. Unpaired T test, ns = P > 0.05. Image orientation: Top is lateral, Right is Apex. Underlying data available in S1 Data. (TIF) [file pbio.3003350.s009.tif]

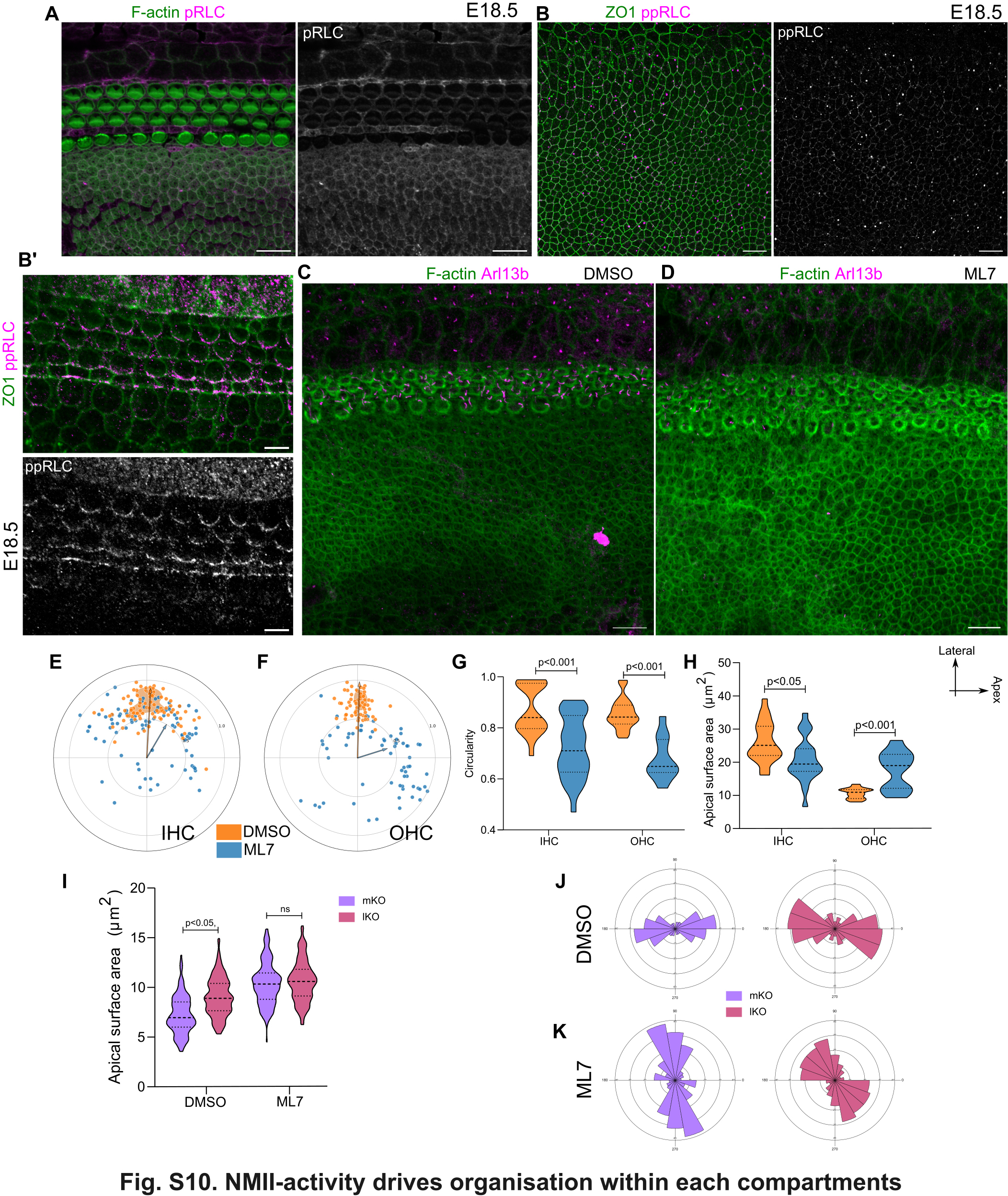

Supplement: S10 Fig — (A) E18.5 OC stained for F-actin (green) and mono-phosphorylated form of RLC (pRLC, magenta, gray). (B) KO and Sensory domain from E18.5 OC stained for ZO1(green) and di-phosphorylated form of RLC (ppRLC, magenta, gray). (C) E16.5 cochlea cultured ex vivo for 8 h in a 3D-collagen droplet culture with DMEM supplemented with DMSO, stained for F-actin (green) and Arl13b (magenta). N = 6 cochlea. (D) E16.5 cochlea cultured ex vivo for 8 h in a 3D-collagen droplet culture with DMEM supplemented with or 25 µM ML7, stained for F-actin (green) and Arl13b (magenta). N = 6 cochlea. (E) Polar coordinates representing position of kinocilia of IHC from OC cultured in presence (blue) or absence (orange) of MLCK-inhibitor ML7. N = 66/68 (DMSO/ML7). (F) Polar coordinates representing position of kinocilia of OHC from OC cultured in presence (blue) or absence (orange) of MLCK-inhibitor ML7. N = 68/61 (DMSO/ML7). (G) Circularity of IHC and OHC from OC cultured in presence (blue) or absence (orange) of MLCK-inhibitor ML7. N = 58/61 for IHC and 69/66 for OHC (DMSO/ML7). (H) Apical surface area of IHC and OHC from OC cultured in presence (blue) or absence (orange) of MLCK-inhibitor ML7. N = 58/61 for IHC and 69/66 for OHC (DMSO/ML7). (I) Apical surface area of mKO and lKO cells from OC cultured in presence or absence of MLCK-inhibitor ML7. N = 193/207 for DMSO and 203/214 for ML7 (DMSO/ML7). (J) Rose stack plot representing the axis of cell elongation for mKO and lKO cells in OC cultured in DMSO. N = 193/207 mKO/lKO. (K) Rose stack plot representing the axis of cell elongation for mKO and lKO cells in OC cultured in ML7. N = 203/214 mKO/lKO. Image orientation: Top is lateral, Right is Apex. Scale Bar: 10 µm. Unpaired T test, ns = non-signficant, P > 0.05. Underlying data available in S1 Data. (TIF) [file pbio.3003350.s010.tif]

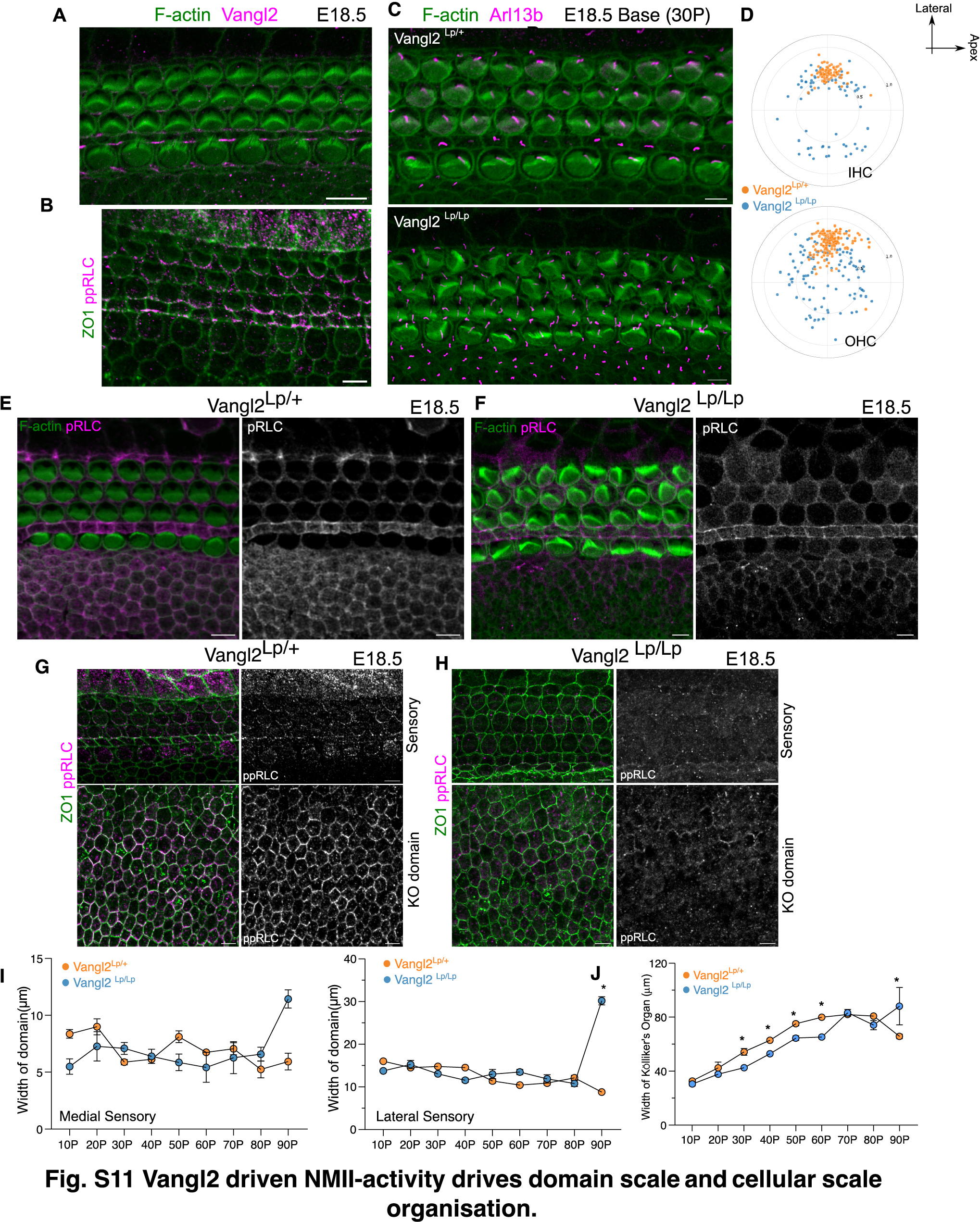

Supplement: S11 Fig — (A) Sensory domain from E18.5 OC stained for F-actin (green) and Vangl2 (magenta). (B) Sensory domain from E18.5 OC stained for ZO1(green) and di-phosphorylated form of RLC (ppRLC, magenta, gray). (C) Base region (30P) of E18.5 OC from heterozygous (Vangl2 Lp/+) and homozygous (Vangl2 Lp/Lp) looptail mutant stained for F-actin (green) and Arl13b (magenta). N = 6 cochlea. (D) Polar coordinates of kinocilia of IHC and OHC at Base region (30P) of E18.5 OC from heterozygous (Vangl2 Lp/+) in orange and homozygous (Vangl2 Lp/Lp) looptail mutant in blue. N = 66/66 for IHC and N = 110/110 for OHC (Het/Homo). (E) E18.5 OC from heterozygous looptail mutant stained for F-actin (green) and pRLC (magenta, gray). (F) E18.5 OC from homozygous looptail mutant stained for F-actin (green) and pRLC (magenta, gray). (G) E18.5 OC from heterozygous looptail mutant stained for ZO1(green) and ppRLC (magenta, gray). (H) E18.5 OC from homozygous looptail mutant stained for ZO1(green) and ppRLC (magenta, gray). (I) Width of medial and lateral sensory domain along the OC from heterozygous (Vangl2 Lp/+) and homozygous (Vangl2 Lp/Lp) looptail mutant at E18.5. N = 4 embryos. (J) Width of Kölliker’s organ along the OC from heterozygous (Vangl2 Lp/+) and homozygous (Vangl2 Lp/Lp) looptail mutant at E18.5. N = 4 embryos. Image orientation: Top is lateral, Right is Apex. Scale Bar: 10 µm. Unpaired T test, ns = non-signficant, P > 0.05, * = P < 0.05. Underlying data available in S1 Data (TIF) [file pbio.3003350.s011.tif]

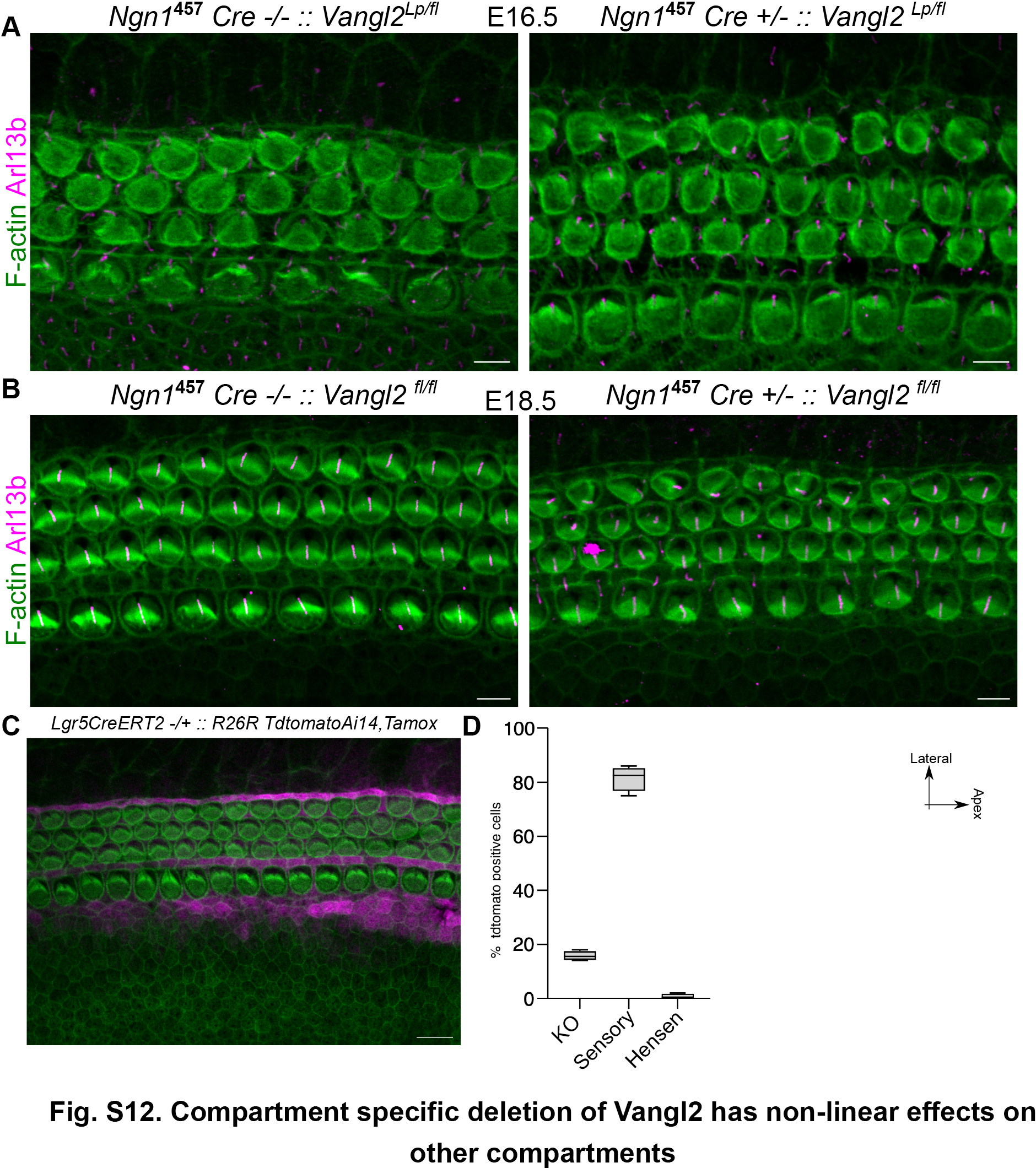

Supplement: S12 Fig — (A) Base region (30P) of E16.5 OC from Cre negative control (Ngn1457-Cre−/−:: Vangl2lp/fl) and Ngn1457cre positive mutant (Ngn1457-Cre+/−:: Vangl2lp/fl) stained for F-actin (green) and Arl13b (magenta). (B) Base region (30P) of E18.5 OC from Cre negative control (Ngn1457-Cre−/−:: Vangl2fl/fl) and Ngn1457cre positive mutant (Ngn1457-Cre+/−:: Vangl2fl/fl) stained for F-actin (green) and Arl13b (magenta). Note: here both copies of Vangl2 is flox allele. (C) E18.5 OC from Lgr5CreERT2::Ai14, induced with Tamoxifen stained with F-actin (green) showing cre-mediated expression of Tdtomato (magenta). (D) Percentage of tdtomato positive cells from the Ngn1457-Cre::Ai14 cochlea in KO, sensory and lateral non-sensory domain. N = 3. Scale Bar: 10 µm. Image orientation: Top is lateral, Right is Apex. Underlying data available in S1 Data. (TIF) [file pbio.3003350.s012.tif]

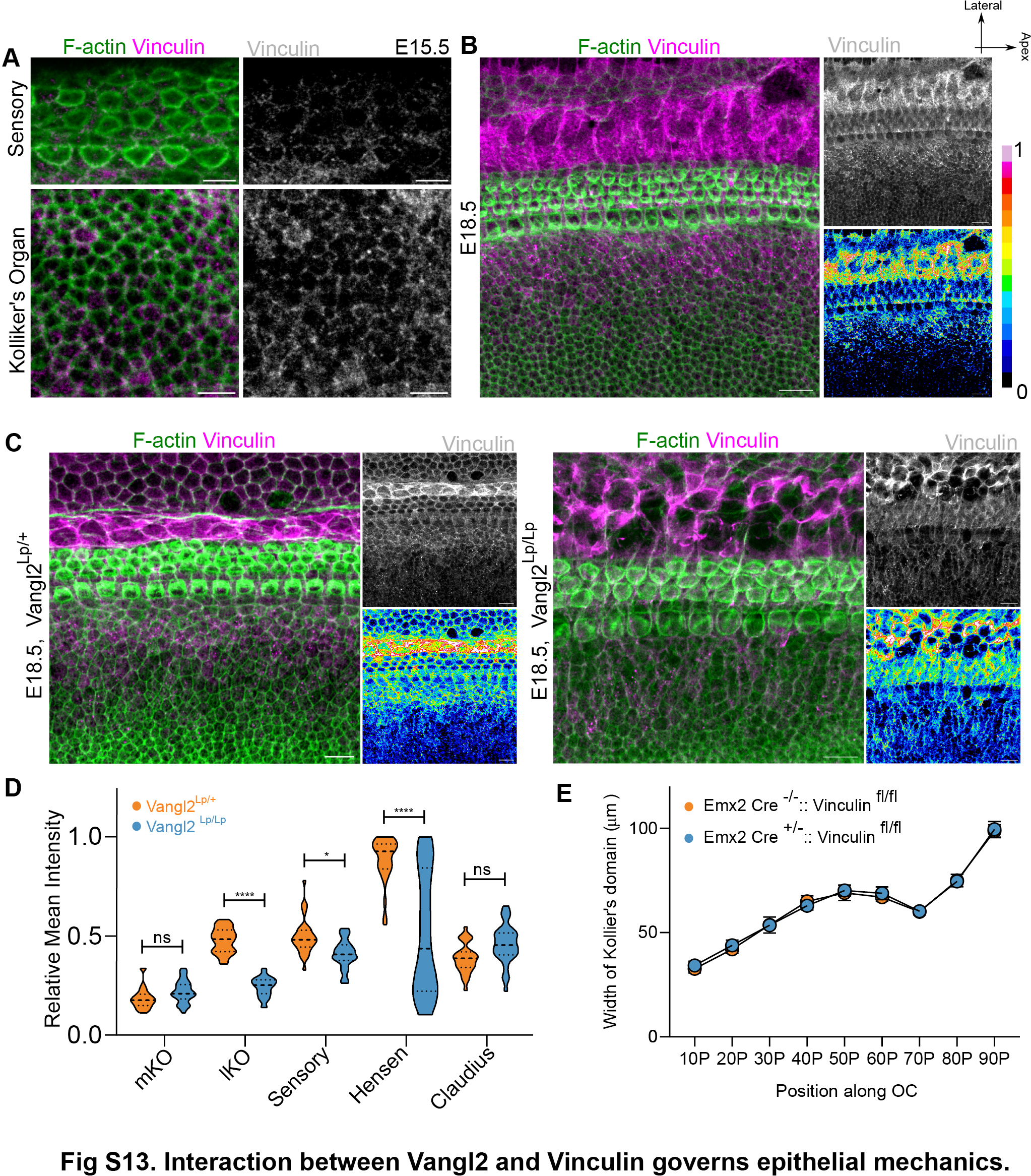

Supplement: S13 Fig — (A) Base region of E15.5 OC stained for F-actin (green) and Vinculin (magenta and gray), showing its localization on cell junctions of sensory and non-sensory compartments. (B) Base region of E18.5 OC stained for F-actin (green) and Vinculin (magenta, gray and heat map) showing higher localization of Vinculin on Hensen’s cells and the lKO cells. (C) Base region of E18.5 OC from heterozygous (Vangl2 Lp/+) and homozygous (Vangl2 Lp/Lp) looptail mutant stained for F-actin (green) and Vinculin (magenta, gray and heat map). (D) Relative Fluorescence Intensity of Vinculin in cells of sensory and non-sensory compartments at E18.5 OC from heterozygous (Vangl2 Lp/+) and homozygous (Vangl2 Lp/Lp) looptail mutant. N = 30 junctions from each cell type and genotype. (E) Width of Kölliker’s organ along the OC from the control (Emx2-Cre−/−:: Vinculin fl/fl) and mutant for vinculin (Emx2-Cre+/−:: Vinculin fl/fl) at E18.5. N = 3 embryos. Scale Bar: 10 µm. Image orientation: Top is lateral, Right is Apex. Unpaired T test, ns = non-signficant, P > 0.05, * = P < 0.05. **** = P < 0.0001. Underlying data available in S1 Data. (TIF) [file pbio.3003350.s013.tif]
