# Supplementary material for: Coupling between spatial compartments integrates morphogenetic patterning in the organ of Corti
Source: PLoS Biol. 2025 Sep 9;23(9):e3003350. doi: 10.1371/journal.pbio.3003350 (PMC12419656; doi:10.1371/journal.pbio.3003350)
Supplement: S1 Table — (PDF) [file pbio.3003350.s014.pdf]

S1 Table

Mouse strains used

Mouse strain	Identifier	Source
Vangl2 (looptail)	JAX:000220	Jackson Laboratory
Vangl2 Flox	JAX: 025174	Michael Deans via Jackson Laboratory [1]
TdtomatoAi14	JAX: 007914	Jackson Laboratory
Fgfr1-Flox	NA	Juha Partanen [2]
Emx2-Cre	NA	Shinichi Aizawa [3]
Sox10-Cre	JAX:025807	William Richardson via Jackson Laboratory [4]
Six1enh21 Cre	NA	Shigeru Sato [5]
Ngn1^{457}-Cre	JAX:012859	Jane Johnson via Jackson Laboratory [6]
Lgr5-IRES-CREERT2	JAX:008875	Hans Cleavers via Jackson Laboratory [7]
Vinculin-Flox	NA	Alice E. Zemljic-Harpf via Srikala Raghavan [8]

References

1. Copley CO, Duncan JS, Liu C, Cheng H, Deans MR. Postnatal refinement of auditory hair cell planar polarity deficits occurs in the absence of Vangl2. J Neurosci. 2013;33(35):14001-16. doi: 10.1523/JNEUROSCI.1307-13.2013. PubMed PMID: 23986237; PubMed Central PMCID: PMCPMC3756750.

2. Pirvola U, Ylikoski J, Trokovic R, Hebert JM, McConnell SK, Partanen J. FGFR1 is required for the development of the auditory sensory epithelium. Neuron. 2002;35(4):671-80. PubMed PMID: 12194867.

3. Kimura J, Suda Y, Kurokawa D, Hossain ZM, Nakamura M, Takahashi M, et al. Emx2 and Pax6 function in cooperation with Otx2 and Otx1 to develop caudal forebrain primordium that includes future archipallium. J Neurosci. 2005;25(21):5097-108. doi: 10.1523/JNEUROSCI.0239-05.2005. PubMed PMID: 15917450.

4. Matsuoka T, Ahlberg PE, Kessaris N, Iannarelli P, Dennehy U, Richardson WD, et al. Neural crest origins of the neck and shoulder. Nature. 2005;436(7049):347-55. doi: 10.1038/nature03837. PubMed PMID: 16034409; PubMed Central PMCID: PMCPMC1352163.

5. Sato S, Ikeda K, Shioi G, Nakao K, Yajima H, Kawakami K. Regulation of Six1 expression by evolutionarily conserved enhancers in tetrapods. Dev Biol. 2012;368(1):95-108. doi: 10.1016/j.ydbio.2012.05.023. PubMed PMID: 22659139.

6. Quinones HI, Savage TK, Battiste J, Johnson JE. Neurogenin 1 (Neurog1) expression in the ventral neural tube is mediated by a distinct enhancer and preferentially marks ventral interneuron lineages. Dev Biol. 2010;340(2):283-92. Epub 20100218. doi: 10.1016/j.ydbio.2010.02.012. PubMed PMID: 20171205; PubMed Central PMCID: PMCPMC2854235.

7. Barker N, van Es JH, Kuipers J, Kujala P, van den Born M, Cozijnsen M, et al. Identification of stem cells in small intestine and colon by marker gene Lgr5. Nature. 2007;449(7165):1003-7. Epub 20071014. doi: 10.1038/nature06196. PubMed PMID: 17934449.

8. Zemljic-Harpf AE, Miller JC, Henderson SA, Wright AT, Manso AM, Elsherif L, et al. Cardiac-myocyte-specific excision of the vinculin gene disrupts cellular junctions, causing sudden death or dilated cardiomyopathy. Mol Cell Biol. 2007;27(21):7522-37. Epub 20070904. doi: 10.1128/MCB.00728-07. PubMed PMID: 17785437; PubMed Central PMCID: PMCPMC2169049.
