# Supplementary material for: Coupling between spatial compartments integrates morphogenetic patterning in the organ of Corti
Source: PLoS Biol. 2025 Sep 9;23(9):e3003350. doi: 10.1371/journal.pbio.3003350 (PMC12419656; doi:10.1371/journal.pbio.3003350)
Supplement: S2 Table — (PDF) [file pbio.3003350.s015.pdf]

S2 Table

Oligonucleotide sequences used for PCR-based genotyping mouse strains

Name	Oligonucleotide Sequence 5'- 3'	Annealing Temperature
Vangl2 Flox FWD	CAGAATCCTCCTGTCCCTGA	59°C
Vangl2 Flox REV	CTCAGCTAAACCACCTCTGC	
Cre FWD	TGCCAGGATCAGGGTTAAAGAT	60°C
Cre REV	AGCTTGCATGATCTCCGGTATT	
FGFR1 FWD	AATAGGTCCCTCGACGGTATC	60°C
FGFR1 REV	CTGGGTCAGTGTGGACAGTGT	
Vinculin FWD	CCTGCGCGGGATTACCTCATTGA	58°C
Vinculin REV	TGCTCACCTGGCCCAAGATTCTT	
TdTomato FWD	GGCATTAAAGCAGCGTATCC	60°C
TdTomato REV	CTGTTCCTGTACGGCATGG	
