# Supplementary material for: Coupling between spatial compartments integrates morphogenetic patterning in the organ of Corti
Source: PLoS Biol. 2025 Sep 9;23(9):e3003350. doi: 10.1371/journal.pbio.3003350 (PMC12419656; doi:10.1371/journal.pbio.3003350)
Supplement: S3 Table — (PDF) [file pbio.3003350.s016.pdf]

S3 Table

Primary and secondary antibodies used with the fixation condition

Antibody	Source	Fixation
Myosin 7a	DSHB 138-1	4% PFA 1 hour RT or 4% PFA overnight
Arl13b	Proteintech 17711-1-AP	4% PFA 1 hour RT or 4% PFA overnight
Beta Spectrin II	BD Biosciences 612562	4% PFA 1 hour RT or 4% PFA overnight
E Cadherin	BD Biosciences 610405	4% PFA 30minutes RT or Glyoxyl fixation 30minutes RT
N Cadherin	BD Biosciences 610920	4% PFA 30minutes RT or Glyoxyl fixation 30minutes RT
E Cadherin	Cell signalling Technology 3295	4% PFA 30minutes RT or Glyoxyl fixation 30minutes RT
P75NTR	Merck AB1554	4% PFA 1 hour RT
BLBP	Abcam ab32423	4% PFA 1 hour RT
Vangl2	Sigma HPA027043	4% PFA 30minutes RT
p-RLC	Cell signalling Technology 3675	4% PFA 30minutes RT
pp-RLC	Cell signalling Technology 3674	4% PFA 30minutes RT and 2%TCA 30minutes on ice
ZO1	Sigma MABT339	4% PFA overnight or 4% PFA 30minutes RT and 2%TCA 30minutes on ice
Nectin 1	MBL 48-12	4% PFA 30minutes RT
Nectin 2	MBL 502-57	4% PFA 30minutes RT
R Cadherin	DSHB MRCD5	4% PFA 30minutes RT or Glyoxyl fixation 30minutes RT
E Cadherin Block	DSHB 7D6	NA
N cadherin Block	DSHB 6B3	NA
Sox2	Invitrogen 14-9811-82	Glyoxyl fixation 30min RT
Phalloidin 488	Invitrogen A12379	4% PFA 1 hour RT to 4% PFA overnight
Phalloidin 568	Invitrogen A12380	4% PFA 1 hour RT to 4% PFA overnight
Goat anti-Rabbit 488	Invitrogen A11034	In all fixation conditions

Goat anti- Rabbit 594	Invitrogen A11037	In all fixation conditions
Goat anti-Rabbit 647	Invitrogen A21244	In all fixation conditions
Goat anti-mouse 488	Invitrogen A-11001	In all fixation conditions
Goat anti-mouse 594	Invitrogen A-11005	In all fixation conditions
Goat anti-mouse 647	Invitrogen A-21235	In all fixation conditions
Goat anti-rat 594	Invitrogen A-11007	In all fixation conditions
