# Supplementary material for: Coupling between spatial compartments integrates morphogenetic patterning in the organ of Corti
Source: PLoS Biol. 2025 Sep 9;23(9):e3003350. doi: 10.1371/journal.pbio.3003350 (PMC12419656; doi:10.1371/journal.pbio.3003350)
Supplement: S4 Table — (PDF) [file pbio.3003350.s017.pdf]

S4 Table

Concentration of small molecule inhibitors used for ex-vivo organ culture

Small Molecule Inhibitor	Identifier	Concentration used
ML7	Sigma 475880	25µM
E cadherin Block	DSHB 7D6	5µl/ml (10µg/ml)
N cadherin Block	DSHB 6B3	5µl/ml (10µg/ml)
Su5402	Calbiochem 572630	25µM
