# Supplementary material for: Coupling between spatial compartments integrates morphogenetic patterning in the organ of Corti
Source: PLoS Biol. 2025 Sep 9;23(9):e3003350. doi: 10.1371/journal.pbio.3003350 (PMC12419656; doi:10.1371/journal.pbio.3003350)
Supplement: Attachment — Submitted filename: Anubhav_PLOS_Bio_comments.pdf [file pbio.3003350.s022.pdf]

Reviewer 1

How part of the epithelium in the inner ear especially the cochlea adopts a sensory fate is unknown. It is clear that there is patterning of the various cell types and this patterning medial to lateral is maintained during convergent extension in the longitudinal direction. This manuscript proposes that specific cell adhesion proteins allow cells in a particular domain to remain together. The authors describe a combinatorial code of adhesion molecules defining specific compartments in the developing cochlea. They further elucidated using Fgfr1 conditional Knockouts that Fgfr1 was essential for maintaining the code for the domains. They further show that Fgfr1 activity is necessary even after cell fate choices are made. Disruption of Cdh signaling with specific blocking antibodies show that these adhesion molecules were necessary for domain segregation and that blocking of specific cadherins led to disruption of particular domains. In an interesting experiment deleting Vangl2 in separate compartments showed that each compartment can influence one another. This is a novel finding, a phenomenon they refer to as "compartment coupling". Testing a possible mechanism the authors propose a mechanical force influence and indeed when Vinculin, a protein required for transmission of forces across junctions, is deleted coupling between compartments is disrupted.

There are many studies describing radial patterning of the cochlea and its dependence on various secreted factors. Not so much is known about the maintenance of patterning during the convergent extension required for the cochlea to reach its full length. This study introduces some new players consisting of planar cell polarity factors as well as cell adhesion molecules. It provides an interesting and new perspective for developmental biologists studying the cochlea.

We thank the reviewer for their encouraging comments on our work. We are pleased that they found the work valuable and novel.

Specific suggestions to enhance clarity:

In Figure 1 panel B which is labeled E15.5 looks much older since at E15.5 the cochlea is not well patterned.

We thank the reviewer for pointing this out, and allowing us to clarify this. Hair cells (HCs) differentiation and maturation shows a gradient along the base to apex of the cochlea (PMID: 11973280, PMID: 15042704, PMID: 33046691). The image in Figure 1B is from the mid-base region of E15.5 embryos where the organ of Corti is most mature. In the revised manuscript we show a micrograph from the base region of E15.5 embryos which provides a better comparison to the OC from E18.5 images.

Make graph on Fig S12 larger i.e. the size of the image panels of the same figure.

In the revised manuscript, we have enlarged the graph in Figure S12D.

Figure 4: truncate panel A and enlarge panel B

In the revised manuscript, we have made suggested changes to Figure 4A and B.

Reviewer 2

This manuscript described pattern formation of developing organ of Corti focusing on Kollikers organ, medial non-sensory domain. They identified that organization of these domains established before the sensory epithelium specification, FGF and PCP signals are required for patterning, domain organization is interconnected, and compartment coupling was mediated via vinculin-dependent junctional mechanics.

The mechanism by which pattern development and coupling through junctional mechanics is interesting and this will be the topic of interest in cell biology. However, there are concerns on this manuscript need to be revised before publication.

We thank the reviewer for the positive evaluation of our manuscript and their constructive feedback.

1. The manuscripts contain many typos and mis-labeling figures which are difficult to understand and interpret. For example, in Figure S3G, lKO region (light brown) was indicated as Cdh 1+2+4 positive but it looked like Cdh-2+4 positive based on other subfigures. Lateral-sensory region (pink) also seem to be labeled by Cdh1+Nectrin1 instead of Cdh2. Lateral non-sensory region (grey) seems Cdh1 only not Cdh1+Nectrin1.

We apologize for our oversight. In the revised manuscript, we have done a thorough check of spelling, grammar, labelling and have improved the readability of our manuscript.

2. In general, the quality of the pictures is not optimal to assess what the authors claim. Major concern is that the phalloidin staining in the KO region, especially mKO region is hard assess (Fig3G, Fig4C, Fig5F, FigS8F). This may be due to flattening the KO region since KO region is composed of multilayer increasing from lKO to mKO.

We thank the reviewer for pointing this out. In the KO region, the nuclei of cells are organised at multiple levels along the apico-basal axis and the tissue shows a curvature along the medio-lateral axis. This does seem to have some impact on the mKO cells when we make 2D-projections from our confocal stacks. This is amplified when the images are reduced in size and resolution during the evaluation process.
We have rectified this by changing images (Fig 3G) and enhancing the resolution (Fig 4). The images in the revised manuscript are now clearer and more accessible.

3. There is not detailed description how to measure axis of elongation for KO cells. This makes it difficult to understand Rose stack plots.

In the revised manuscript, we have incorporated a schematic in Figure S8 to represent the measurement. A more detailed description of the mKO and lKO measurements has been included in the method section. Briefly, we segment the cell boundaries using F-actin staining and calculate the Feret angle (long-axis of the cells) with respect to the proximal-distal (base to apex of cochlea) axis of the image. We plot the distribution of these angles in a rose-stack graph, where 90° is perpendicular to the base-apex of the cochlea.

4. In Figure2, it is difficult to assess the expression pattern of Cdh1, 2 and Phalloidin. Inner hair cells are supposed to specify at E14.5 by expressing Atoh1 and Sox2, Jag1, and are supposed to be expressed in the organ of Corti. It would be better to co-label any of those with phalloidin and cadherins to visualize sensory patch. Cdh2 staining in panel G was not convincing as plotted on the RFI on panel H. The authors said "the patterned expression of Cdh1 and Cdh2 OC was absent" in Fgfr1-cko. This statement needs to be more specific. Are Cdh1 and Cdh2 expressed in the same cells or in a mosaic pattern? Since Chd1 and Chd2 are not co-expressed in the control OC, this would be important to know the exact expression pattern.

In our analysis, Cdh1, Cdh2 and Cdh4 expression in the Organ of Corti (OC) is localised to the junctions. We further quantified intensity is along the medio-lateral axis in Figure 2E. The loss in resolution after compressing images has introduced some issues.

Following the suggestion from the reviewer, we have co-labelled Cdh1 with Sox2, to confirm the localisation of cadherin molecules with respect to the pro-sensory region (Figure S3H). In the FGF mutants, the restricted expression of Cdh2 to the medial edge of the OC is lost. We observe that both Cdh1 and Cdh2 are expressed in all cells along the medio-lateral axis of OC. In the revised manuscript, we provide a clearer image to support our claim, along with the intensity quantification in Figure 2H. We have clarified our text to describe this result.

5. In Figure 3G, scale of control and Vangl2Lp/Lp panel is different. This need to be fixed to for readers to compare easily the area of cells.

We thank the reviewer: in the revised manuscript we have changed this image for better comparison with the control samples.

6. Data in Figure 6 is surprising. Only 10% of cells within the KO domain were targeted in Ngn1-457Cre. Yet, degree of the phenotype in Vangl2-cKO are comparable to Vangl2Lp/Lp and Vangl2-Lgr5-cKO. This is dramatic effect of Vangl2 in patterning of OC. Authors need to discuss this phenomenon.

We apologise for the lack of clarity in our writing. The Vangl2-Ngn1 CKO does show a milder phenotype in the sensory compartment, then the Vangl2Lp/Lp or the Vangl2-Lgr5-cKO. What is surprising is that Ngn1-Cre is not expressed in the sensory domain, and the defect in polarity (of OHC3) is at a considerable distance from the sites of Vangl2 deletion. This is a surprising result, and is not a non-cell autonomous domineering effect of Vangl2, which occurs more locally around the sites of deletion. This forms part of the basis for compartment coupling in the organ of Corti. Vangl2 affects tissue mechanics, which affect the interactions between compartments. This is manifest as defects in OHC3. We have made this clearer in our writing.

> *7. The phenotype of Vinclulin-cKO is different from Vangl2-KO, increased IKO surface area instead of decrease and different mKO long axis direction. Authors need to explain this difference.*

Vinculin is critical for the force transmission across cells. Although the localisation of Vinculin localisation has been shown to be influenced by planar cell polarity cues and protein tyrosine kinase 7 (Ptk7) in OC (11). However it does seem to integrate tension from both contractility and adhesion. We show that while heterogenous junctional contractility is perturbed in Vangl2 mutants, adhesion, the adhesion code is maintained. Thus, the difference in phenotype of KO cells in Vangl2 KO and Vinculin cKO may arise from one or both of the following possibilities:

1. In the Vangl2 KO (looptail) the spiral shape of the cochlea is perturbed, however in the Vinculin cKO it is maintained. The difference in size and shape of KO cells could arise, in part, from this difference in global shear.
2. Vangl2 KO decreases the contractility of cells by decreasing the di-phosphorylation status of non-muscle myosin II (Figure S10). Vinculin is known to integrate information of adhesion and contractility at the apical junctions and focal adhesion sites at the basement membrane. Hence, the phenotype observed in Vinculin cKO is a combinatorial effect of decreased communication of adhesion, contractility and basement membrane interaction, while the phenotype in Vangl2 KO is driven by reduced contractility.

Following the suggestion from the reviewer, we discuss this in our results and the discussion section of the manuscript.

Reviewer 3

> *The findings presented in this study show that cell patterning in the developing mouse cochlea is associated with a combinatorial expression of adhesion molecules, which segregates the OC into spatially defined compartments and allow planar cell polarity (PCP) cues to regulate cell organisation within each compartment. The work implicates compartment coupling as a major determining factor in cochlear morphogenesis. Although we understand a significant*

amount about how the cells within the cochlear epithelium are specified, the mechanisms underlying its growth and morphogenesis are less clear. This is therefore a novel study that addresses an under explored and important aspect of inner ear development.

The authors use different mouse mutants to understand how compartment-coupling and compartment integrity is maintained during convergent extension in the developing cochlea. They describe compartment-coupling as mechanism where a non-linear influence on the organisation in one compartment can disrupt cellular organisation and patterning in another. Overall, this compartment-coupling is regulated by PCP signalling via Vangl2.

Given that Vangl2 regulates junctional mechanics between different cells, the authors explored a potential mechanical component of compartment coupling.

We thank the reviewer for their positive evaluation of our manuscript and the constructive feedback.

Questions to authors:

The authors describe a change in cell shape index and circularity in the Ffgr1 mutants. Do the authors think this could be due to changes in the cytoskeleton or cell-specific tension? Were either of these parameters investigated or compared between WT and mutant cochleae?

During cochlear morphogenesis, cell shape is regulated by intrinsic cytoskeletal organization, differential interfacial tension, and extrinsic forces imposed by neighboring compartments and global tissue architecture. In *Fgfr1* mutants, we observe disrupted expression of adhesion molecules, indicative of altered mechanical tension at cellular interfaces. This aligns with prior studies on adhesion molecule mutants, such as *Nectin*, which demonstrated changes in cell shape index and circularity, emphasising the idea that cell-specific tension contributes to morphogenetic shape changes. However, we cannot exclude that *Fgf* signaling may also directly influence the cytoskeleton (PMID: 18583133), and thus may have a role in cell shape. To determine this, we are establishing a synthetic biology-based approach to dissect the contributions of adhesion-mediated tension versus cytoskeletal remodeling. These efforts are ongoing and may form the basis of a future manuscript.

Would it be possible to infer any hypothesis about how epithelial mechanics might between cochlear compartments?

We agree with the reviewer that epithelial morphogenesis is different across compartments within the cochlea. The sensory domain consists of two distinct compartments: the medial sensory compartment, which gives rise to inner hair cells (IHCs), and the lateral sensory compartment, which gives rise to outer hair cells (OHCs). In the lateral sensory compartment, morphogenesis is characterized by differential tension at hair cell–supporting cell (HC–SC) and supporting cell–supporting cell (SC–SC) junctions. This tension is governed by a

combination of adhesion, particularly through Nectin-mediated interactions (PMID: 21798896), and contractility, as indicated by the phosphorylation of regulatory light chain (RLC) (PMID: 40280944). Together, these forces, together with a shear-induced interaction with the lateral non-sensory domain, contribute to the establishment of a highly ordered hexagonal packing of OHCs (PMID: 33046691). This organization is further supported by an increase in apical surface area of HCs and a quasi-stratified arrangement of HC and SC nuclei (PMID: 28870992). In this compartment, spatial and orientational order emerges through local intercalation events, guided in part by global tissue-level cues.

In contrast, the medial sensory compartment, which will give rise to the IHC and associated SC, forms a single row of cells, and is organised through an interesting, three-dimensional, two-step intercalation process known as "hopping intercalation," (PMID: 36812313). Our data (Fig. 2) reveal distinct adhesion profiles in this compartment, including specific expression of Nectin-1. Despite sharing a common lineage with the lateral sensory compartment (PMID: 24465223, PMID: 22235191), the medial compartment follows a morphogenetic path that is mechanistically distinct.

The KO compartment is interesting. The cells are undergoing cellular organisation driven primarily by cell intercalation (Figure 3B and S9E). However we also observe an increase in the apical surface area and change in the axis of elongation of lKO cells, that is driven by mechanics in the sensory domain, as evidenced by both the sensory domain specific of Vangl2 and the deletion of Vinculin. We can hypothesise that both the CE movements of the KO and the change in aspect ratio and surface area of the lKO, may drive a more curvilinear type of cell flow in the KO. We are currently working with theory colleagues to formalise these ideas, and design experiments to test this.

This comment from the reviewer is very thoughtful and we have included this in our discussion.

Are there differences in the expression levels of vinculin in different compartments?

Following suggestion from the reviewer we stained OC for Vinculin (Figure S13). We observed vinculin at all junctions, but see higher expression in cells at the interface of sensory domain, namely: Hensen's cells and lKO cells.

The differences in the direction of the long axis of lKO and mKO cells are reduced in Emx2-Cre+/- ::Vincfl/f 'These data suggest the coupling between compartments is mediated, at least in part, through mechanics.'
From this statement, would the hypothesis assume that cells are smaller because the are under less tension, or because they are being compressed from changes in cell properties/behaviour in another compartment where vinculin activity has been lost?
Were there any differences in gross cochlear length in the absence of vinculin?

lKO cells form at the interface of the medial sensory compartment and KO. This interface is under greater tension, both from the shear force generated by intercalation of cells in the medial sensory domain, and from interfacial tension generated at the boundary between two adhesion domains. We think that this increased tension drives the shape change of the lKO cells so that their long edges are aligned with the medial sensory domain. In Vangl2-Lgr5-CKO, the intercalation in the medial sensory compartment is perturbed, and results in a perturbation of lKO cells. The reviewer is correct: the absence of vinculin would dampen the transmission of shear force, and to some extent interfacial tension, across compartments. Our new data finds that vinculin is enriched in lKO junctions. This is consistent with the view of the compartment interface as being under tension; as vinculin localisation increases in junctions that are under tension.

We have not observed a significant difference in cochlear length in the absence of vinculin.

> *Were there any differences in the OC width? If mechanical coupling has been altered, one could hypothesise potential changes in compartment size. Was this looked at?*

We thank the reviewer for this suggestion. We re-analysed our data and found that the width of the KO domain in the Vinculin mutants are comparable to that observed in control samples.

> *Is it possible to provide data on normal expression of vinculin in the developing cochlea?*

The antibody for Vinculin (Sigma V9131) used by Lee et al (PMID: 22560610) has shown expression in the sensory domain. Using the same antibody we show the expression of Vinculin in the sensory and the non-sensory domains during development in Figure S13.

> *The authors describe a loss of circularity in a decrease in apical surface area in OHCs following treatment with ML7(Fig. S10C-H), This finding is attributed to a decrease in spatial organisation within the sensory domain. How can the authors be sure this is not simply a reduction in overall HC growth or developmental progression?*

At E14.5 newly born HCs have smaller apical surface area with a cilium closer to the centroid. Over the next 2 days, the cilia moves to an eccentric position (preprint version 1 of this manuscript, PMID: 36812313).Using a mild inhibition of MLCK, ML7, we show the apical surface area of OHC increases and that of IHC decreases compared to the control. We note that the cilia in both IHCs and OHCs are eccentric, suggesting a limited influence of ML7 inhibition on developmental progression.

Previously, Driver et al (PMID: 28870992) have shown the increase in HC volume is a week-long process. They show a potent inhibition of myosin by blebbistatin at the time scale of hours leads to an increase in apical surface area compensated by decrease in HC height,

preserving volume. This suggests that spatial organisation on the apical surface has a contribution from the three-dimensional organisation of the epithelium. Over the timescale of the experiment, the mild inhibition by ML7 for 6 hours would have a limited effect on the growth of HC, but we still show defects in spatial organisation.

Given the reported role for vinculin in compartment coupling do the authors think there is any interaction between Vangl2 and vinculin in terms of regulating cell behaviour or cell tension across different compartments. What would be the predicted outcome of reduced vinculin in a Vangl2 LoF model? How would this impact compartment coupling? Could the authors provide any comment on this?

Previously Lee et al (PMID: 22560610) have shown PCP protein Frizzled and protein tyrosine kinase 7 regulates the localisation of Vinculin in cochlear HCs. In Figure S13, we show the Vangl2 looptail mutants (LOF model) misaligns the localisation of Vinculin on the junctions. This suggests a possible biochemical interaction between Vangl2 and Vinculin. However, as Vangl2 regulates junctional tension and Vinculin localisation is dependent on junctional tension, a possible feedback through mechanics is also possible. In the revised manuscript, we include this point in our discussion.

We and others have shown the role of junctional mechanics in development of OC, we speculate that reduced Vinculin in the Vangl2 mutants would aggravate the defects in polarity and spatial organisation of HCs. In the inner ear specific mutants of Vangl2, Copley et al have shown the refinement of HC polarity during the postnatal development, possibly through junctional remodelling. In this context, the reduced vinculin in Vangl2 mutants would impair the refinement of HC polarity.

In Figure 5A the hair cells in the Lgr5CreERT2 +/-:: Vangl2Lp/fl::Tamox explants appear larger. Is this a consistent observation?

The apical surface area of HCs in the Lgr5CreERT2 +/-:: Vangl2Lp/fl::Tamox mutants do show variability between samples and between adjacent cells. As this is a tamoxifen inducible mutant, this variability could result from differences in cre activity, as well as the non-cell autonomous role of Vangl2. We show this variation in the graph figure 5D, E.

'Moreover, mutant cochlea showed no difference between the direction of the long axis of lKO cells (mediolateral to the tissue axis) and that of the mKO cells (parallel to the tissue axis) (Fig. 5F, H)'

In Figure 5F the directionality/orientation of the KO cells appears different along the medial-to-lateral axis in the Lgr5CreERT2 +/-:: Vangl2Lp/fl::Tamox compared to Lgr5CreERT2 +/-:: Vangl2Lp/fl::Corn oil. The cells in Lgr5CreERT2 +/-:: Vangl2Lp/fl::Corn oil appear polarised along the basal-to-apical axis whereas those in the Lgr5CreERT2 +/-:: Vangl2Lp/fl::Tamox appear polarised along medial-to-lateral axis. Can the authors provide further clarification on this?

'This data suggests a reciprocal influence of the sensory domain on cellular organisation within the KO compartment (Fig. 5I)………'
Could the authors provide further explanation on how they arrived at this conclusion. It is not immediately clear from the current text.

The reviewer is correct. We also want to state that the difference in the axis of elongation in controls is lost in the mutants. This loss of organisation in the KO compartment, when Vangl2 is deleted from the sensory domain suggests a inter-compartment effect of Vangl2. This is similar to the Vangl2 Ngn1-457 cre deletion, where an effect on OHC3 polarity is observed in lateral sensory compartment. We have clarified our text to represent this better.

Could the authors comment on whether any differences in total cochlear length were observed between Lgr5CreERT2 +/-:: Vangl2Lp/fl::Corn oil and Lgr5CreERT2 +/-:: Vangl2Lp/fl::Tamox?

In our observations, we did not find any difference in cochlear length in the Lgr5CreERT2 +/-:: Vangl2Lp/fl::Tamox mutants. This is consistent with data from the inner ear specific deletions of Vangl2 (driven by Pax2) (PMID: 23986237), which shows comparable cochlear length in conditional deletions and wild types.

Can the authors provide any insight as to how compartment coupling might regulate gross cochlear morphogenesis or epithelial bending? How might coordinated signalling between Fgf and ERK described by Ishii et al in 2020 be impacted in the different compartment-specific Vangl2 LoF cochleae?

This is an excellent point, and we thank the reviewer for prompting this reflection. Ishii et al., together with the work from the Sprinzak group are seminal studies in understanding the cell biology behind spiralling of the cochlea, and how pattern may emerge from the cell flows in the epithelium. Ishii describe two types of cell behaviours that contribute to bending; luminal nuclear stalling in the medial side of the epithelium and helical cell flows reciprocal to the FGF-ERK waves originating at the tip of the duct. Both behaviours establish stresses that need to be dissipated laterally. Here, we will indulge in some speculation - Vangl2 localisation is driven in part by mechanical tension, and it is possible that this localisation generates patterns of contractility that effectively dissipate the tension. Compartment-specific mutations in Vangl2 could disrupt this mechanical feedback resulting in altered force patterns. As proposed by Ishii et al., such disruptions would impair mechano-chemical feedback loops, including FGF-ERK signaling, thereby compromising coordinated cochlear morphogenesis. Surprisingly, the compartment specific Vangl2 mutants do not appear to affect spiralling: however we note that an inner ear epithelium specific KO of Vangl2 does not lead to defects in the length of the cochlea.

Similarly, how might cellular organisation within compartments be impacted following loss of Shh signalling or in other CE mutants not associated with components the core PCP pathway?

During morphogenesis of cochlea, the Shh signalling is known to drive cell specification in cochlea. The mutants of Shh, its receptor and the downstream effector have shown defects in differentiation of HCs, cochlear roof and lateral non-sensory cells as well as showed reduced proliferation (Son et al 2015, Qin et al 2024). As proposed by Ishii et al, the bending of cochlea requires a cell flow from lateral edge and epithelial roof, the disruption of their differentiation would impair the movement leading to defects in convergent extension of OC. In mutants of shh and several (PCP independent) molecules, the defects in the convergent extension of OC results in accumulation of HCs at the apical end with defects in spatial organisation and planar polarity. Hence, these polarity defects could be associated with an impaired mechano-chemical feedback at global scale (Ishii et al) or disruption in force patterning in the sensory domain (Prakash et al 2025).

Minor points:

Check that all referencing is presented in the same format. Some references are 'numbered' and some refer to the author names. Huh et al 2011 and Ono et al 2014 are missing from the bibliography.

I recommend a thorough check of the final document for any remaining minor spelling/typographical errors.

We have done a thorough check for spelling and typographical errors and amended our text to enhance readability.
