# Supplementary material for: Coupling between spatial compartments integrates morphogenetic patterning in the organ of Corti
Source: PLoS Biol. 2025 Sep 9;23(9):e3003350. doi: 10.1371/journal.pbio.3003350 (PMC12419656; doi:10.1371/journal.pbio.3003350)
Supplement: Attachment — Submitted filename: Comments to Authors_PlosBiology.docx [file pbio.3003350.s020.docx]

The findings presented in this study show that cell patterning in the developing mouse cochlea is associated with a combinatorial expression of adhesion molecules, which segregates the OC into spatially defined compartments and allow planar cell polarity (PCP) cues to regulate cell organisation within each compartment. The work implicates compartment coupling as a major determining factor in cochlear morphogenesis. Although we understand a significant amount about how the cells within the cochlear epithelium are specified, the mechanisms underlying its growth and morphogenesis are less clear. This is therefore a novel study that addresses an under explored and important aspect of inner ear development.

The authors use different mouse mutants to understand how compartment-coupling and compartment integrity is maintained during convergent extension in the developing cochlea. They describe compartment-coupling as mechanism where a non-linear influence on the organisation in one compartment can disrupt cellular organisation and patterning in another. Overall, this compartment-coupling is regulated by PCP signalling via Vangl2.

Given that Vangl2 regulates junctional mechanics between different cells, the authors explored a potential mechanical component of compartment coupling.

**Questions to authors:**

The authors describe a change in cell shape index and circularity in the Ffgr1 mutants. Do the authors think this could be due to changes in the cytoskeleton or cell-specific tension? Were either of these parameters investigated or compared between WT and mutant cochleae? Would it be possible to infer any hypothesis about how epithelial mechanics might between cochlear compartments? Are there differences in the expression levels of vinculin in different compartments?

*The differences in the direction of the long axis of lKO and mKO cells are reduced in Emx2-Cre+/- ::Vincfl/f ‘These data suggest the coupling between compartments is mediated, at least in part, through mechanics.’*

From this statement, would the hypothesis assume that cells are smaller because the are under less tension, or because they are being compressed from changes in cell properties/behaviour in another compartment where vinculin activity has been lost?

Were there any differences in gross cochlear length in the absence of vinculin?

Were there any differences in the OC width? If mechanical coupling has been altered, one could hypothesise potential changes in compartment size. Was this looked at?

Is it possible to provide data on normal expression of vinculin in the developing cochlea?

The authors describe a loss of circularity in a decrease in apical surface area in OHCs following treatment with ML7(Fig. S10C-H), This finding is attributed to a decrease in spatial organisation within the sensory domain. How can the authors be sure this is not simply a reduction in overall HC growth or developmental progression?

Given the reported role for vinculin in compartment coupling do the authors think there is any interaction between Vangl2 and vinculin in terms of regulating cell behaviour or cell tension across different compartments. What would be the predicted outcome of reduced vinculin in a Vangl2 LoF model? How would this impact compartment coupling? Could the authors provide any comment on this?

In Figure 5A the hair cells in the *Lgr5CreERT2 +/-:: Vangl2Lp/fl::Tamox* explants appear larger. Is this a consistent observation?

*‘Moreover, mutant cochlea showed no difference between the direction of the long axis of lKO cells (mediolateral to the tissue axis) and that of the mKO cells (parallel to the tissue axis) (Fig. 5F, H)’*

In Figure 5F the directionality/orientation of the KO cells appears different along the medial-to-lateral axis in the *Lgr5CreERT2 +/-:: Vangl2Lp/fl::Tamox* compared to *Lgr5CreERT2 +/-:: Vangl2Lp/fl::Corn oil.* The cells in *Lgr5CreERT2 +/-:: Vangl2Lp/fl::Corn oil* appear polarised along the basal-to-apical axis whereas those in the *Lgr5CreERT2 +/-:: Vangl2Lp/fl::Tamox* appear polarised along medial-to-lateral axis. Can the authors provide further clarification on this?

*‘This data suggests a reciprocal influence of the sensory domain on cellular organisation within the KO compartment (Fig. 5I)………’*

Could the authors provide further explanation on how they arrived at this conclusion. It is not immediately clear from the current text.

Could the authors comment on whether any differences in total cochlear length were observed between  *Lgr5CreERT2 +/-:: Vangl2Lp/fl::Corn oil* and *Lgr5CreERT2 +/-:: Vangl2Lp/fl::Tamox?*

Can the authors provide any insight as to how compartment coupling might regulate gross cochlear morphogenesis or epithelial bending? How might coordinated signalling between Fgf and ERK described by Ishii et al in 2020 be impacted in the different compartment-specific Vangl2 LoF cochleae?

Similarly, how might cellular organisation within compartments be impacted following loss of Shh signalling or in other CE mutants not associated with components the core PCP pathway?

**Minor points:**

Check that all referencing is presented in the same format. Some references are ‘numbered’ and some refer to the author names. Huh et al 2011 and Ono et al 2014 are missing from the bibliography.

I recommend a thorough check of the final document for any remaining minor spelling/typographical errors.
